# Footprints of Immune Cells in the Pancreas in Type 1 Diabetes; to “B” or Not to “B”: Is That Still the Question?

**DOI:** 10.3389/fendo.2021.617437

**Published:** 2021-02-25

**Authors:** Pia Leete, Noel G. Morgan

**Affiliations:** Exeter Centre for Excellence in Diabetes (EXCEED), University of Exeter Medical School, Exeter, United Kingdom

**Keywords:** CD20+ B-lymphocyte, CD8+ T-lymphocyte (CTL), insulitis, islet, inflammation

## Abstract

Significant progress has been made in understanding the phenotypes of circulating immune cell sub-populations in human type 1 diabetes but much less is known about the equivalent populations that infiltrate the islets to cause beta-cell loss. In particular, considerable uncertainties remain about the phenotype and role of B-lymphocytes in the pancreas. This gap in understanding reflects both the difficulty in accessing the gland to study islet inflammation during disease progression and the fact that the number and proportion of islet-associated B-lymphocytes varies significantly according to the disease endotype. In very young children (especially those <7 years at onset) pancreatic islets are infiltrated by both CD8+ T- and CD20+ B-lymphocytes in roughly equal proportions but it is widely held that the CD8+ T-lymphocytes are responsible for driving beta-cell toxicity. By contrast, the role played by B-lymphocytes remains enigmatic. This is compounded by the fact that, in older children and teenagers (those ≥13 years at diagnosis) the proportion of B-lymphocytes found in association with inflamed islets is much reduced by comparison with those who are younger at diagnosis (reflecting two endotypes of disease) whereas CD8+ T-lymphocytes form the predominant population in both groups. In the present paper, we review the current state of understanding and develop a proposal to stimulate further discussion of the roles played by islet-associated B-lymphocytes in human type 1 diabetes. We cite evidence indicating that sites of direct contact can be found between CD8+ and CD20+-lymphocytes in and around inflamed islets and propose that such interactions may be important in determining the efficiency of beta cell killing.

## Introduction

The infiltration of immune cells into the pancreases of people with type 1 diabetes was described in graphic detail more than a century ago by pioneers such as Weichselbaum (1) and Schmidt (2), whose elegant drawings revealed the structure of the islets and their attendant infiltrate. In particular, they drew attention to the presence of unidentified groups of very small cells (almost certainly immune cells) adjacent to the islets and, as such, provided an early representation of the process subsequently termed “insulitis” by von Meyenberg (3). Such careful observations and exquisite attention to detail paved the way for subsequent generations of investigators to apply ever more sophisticated technologies [including, for example, the latest methods of multiplexed *in situ* imaging mass cytometry (4, 5)] as a means to confirm and extend the original observations. As a consequence, it might be reasonably expected that, by now, an essentially complete picture would have emerged to describe and explain the involvement of islet-infiltrating immune cells in type 1 diabetes. However, it is salutary to reflect that, at a time when personalized immunotherapy is increasingly mooted as the means by which the progression of type 1 diabetes will soon be stalled in susceptible individuals (6, 7), we still have only a rudimentary understanding of the immune processes occurring in islets as the disease develops.

There are many reasons for this continued lack of understanding including the fact that the number of individual cases in which the events have been studied first-hand at, or very soon after, diagnosis remains extremely small (8) and that the islets of Langerhans are inaccessible in living subjects. All of which means that our grasp of the immune processes that culminate in beta cell death in type 1 diabetes are still based as much on surmise and speculation as on hard evidence. Thankfully, some progress has been made and we focus here on one particular aspect of the immunopathology of human type 1 diabetes which remains intriguing and enigmatic, namely a possible role for B-lymphocytes in driving disease. Facets of this subject have been reviewed very effectively by others (9–13), especially in relation to the differing populations of B-lymphocytes found in the circulation and, accordingly, we have concentrated on their roles in the pancreas.

It is non-contentious to argue that B-lymphocytes are involved in the progression of type 1 diabetes since the majority of subjects produce autoantibodies to one or more islet proteins as a hallmark of the disease. However, there are caveats to this scenario since it is often felt that, while B-lymphocytes are involved, they probably occupy little more than a “bit part” role. Support for this notion comes, for example, from isolated case reports such as that documenting the development of type 1 diabetes in an individual with X-linked agammaglobulinemia (14). Because of his underlying condition, the proband cited in this study had very few, if any, circulating B-lymphocytes and no islet autoantibodies but had still developed type 1 diabetes by his mid-teenage years. Hence, the secretion of islet autoantibodies by B-lymphocytes is often seen as a marker of disease rather than a causative factor [i.e., it is “smoke rather than fire” (11)] and their role is considered ancillary. This view contrasts with evidence from the NOD mouse model of type 1 diabetes where the systemic loss of B-lymphocytes has more profound effects and can prevent disease development (15). However, such evidence is easily downplayed by the understanding that disease presentation in NOD mice may not be fully representative of the human condition.

Based on such considerations, there is a temptation to dismiss any fundamental contribution of B-lymphocytes in the progression of type 1 diabetes and to consign them to largely secondary systemic actions associated with autoantibody production. However, this might prove to be an over-simplification and, as a community of type 1 diabetes researchers, we may downplay the role of B-lymphocytes at our peril.

### Where Do B-Lymphocytes Exert Most Influence in Type 1 Diabetes?

As already noted, the fact that B-lymphocytes are responsible for autoantibody production in type 1 diabetes places them in one particular niche; namely the bloodstream. It is here that clonally expanded populations of B-lymphocytes produce and secrete autoantibodies which are targeted to particular epitopes located on a specific sub-group of islet proteins. However, not all B-lymphocytes are circulating, nor are they necessarily engaged actively in antibody secretion since some may play additional roles in the autoimmune process (12).

B-lymphocytes are also found within secondary lymphoid organs such as the draining lymph nodes located in most organs and tissues, and it is here that lymphocyte subpopulations typically engage with one another to prime the autoimmune response (16). The pancreas contains a relative abundance of such lymphoid tissue [more than 50 pancreatic lymph nodes have been reported in some individuals (17, 18)] but, despite this, pancreatic lymph nodes have been analyzed in detail only rarely in human subjects with type 1 diabetes. One of the few such studies examined a selection of cases held within the Network of Pancreatic Organ Donors (nPOD) Biobank and concluded that no clear structural differences exist between the pancreatic lymph nodes found in donors with type 1 diabetes and those without (19). Our own recent collaborative work has also addressed this question and we observed changes indicative of disease associated events (18). These differing conclusions may reflect the varying disease duration among the subjects studied.

In humans, lymph nodes comprise an outer capsule encompassing a cortex of B- and T-cell follicles (arranged in outer and inner zones respectively) displayed around an inner medulla and hilum; the latter serving as a conduit for the egress of lymphatic fluid (18). B-cell follicles localized within the cortex can exist in one of two principal states, being either primary or secondary, with the latter containing the germinal centers in which B-lymphocytes encounter their cognate antigen and are induced to differentiate into plasma cells. We noted that the structural organization of B-cell follicles was atypical in people newly diagnosed with type 1 diabetes when compared to similarly aged control subjects (18). The frequency of B-cell follicles in which germinal centers could be identified, was reduced by a mean of four-fold in recent-onset type 1 diabetes although, intriguingly, this difference was lost as disease duration increased. The reduction in B-cell follicle frequency did not correlate with the “intensity” of insulitis nor with the age at onset of disease, suggesting that this feature may be a characteristic of the early phase of the disease in all subjects. The precise significance of these findings has not been deduced but it is conceivable that the reduction in secondary follicle and germinal center formation may reflect the diversion of large numbers of B-lymphocytes toward a plasma cell phenotype early in disease progression. This would be consistent with our observation that follicle numbers recover at later times since the production of autoantibodies tends to decline with disease duration.

There is an important additional site at which B-lymphocytes reside in type 1 diabetes and this is among the immune cell population associated with inflamed islets (20, 21). The weight of evidence suggests that B-lymphocytes are unlikely to accumulate here as a result of (or to facilitate) increased autoantibody production, implying an alternative role. Thus, we have explored their localization in the islet infiltrates in more detail.

### Islet Infiltrates Contain Varying Proportions of B-Lymphocytes

In order to study B-lymphocyte infiltration in the pancreas in type 1 diabetes we have exploited a collection of autopsy samples held within the Exeter Archival Diabetes Biobank (EADB) which comprises a large number of samples recovered from subjects newly diagnosed with the disease (8). These represent a historical collection but they offer the advantage that, included among them, are samples from very young children (more than 50 are available from children who were under 10years at diagnosis) as well as from those who were older at onset (principally in their teenage years). The samples have been preserved effectively and they allow comparisons to be made of the profiles of insulitis according to age at diagnosis and severity of beta-cell loss in subjects with relatively short disease duration, where active disease is still evident. The collection also contains samples from people who have lived with the disease for longer periods and, in whom, beta-cell destruction is essentially complete.

An important additional feature is that many of the recent-onset pancreas samples available in the EADB contain islets at varying stages of autoimmune attack (21, 22). Thus, some islets have a relatively heavy immune cell infiltrate coupled with extensive beta-cell loss. By contrast, others appear essentially untouched, being devoid of immune infiltrates and having a full complement of beta cells. Still others display an intermediate phenotype such that varying stages of beta cell destruction can be seen in parallel with the presence of small numbers of influent immune cells. By analyzing multiple islets across a series of individual cases at each stage of the process it is possible to gain an impression of the dynamics of immune cell recruitment (and subsequent egress) and of beta cell loss by reconstructing the profile of these processes in pseudotime.

We first undertook such an analysis more than 10 years ago (21) and were surprised to find that the proportion of B-lymphocytes present within islet infiltrates varies according to the stage of islet destruction. Moreover, it also varies in parallel with the number of CD8+ T-cells but not in proportion to the numbers of CD4+ cells present. In all cases studied, and in common with earlier findings (23) it was clear that CD8+ T-cells form the predominant population as insulitis develops. We also noted that the influx of B-lymphocytes closely mirrors the CD8+ cell profile. This could be taken to imply that a similar cocktail of chemokines is involved in recruitment of both cell types but this begs the question as to why B-lymphocytes are responsive to the chemoattractant gradient and what their role at the site might be. As noted above, one possibility is that they enter the islet infiltrates as antibody producers but, arguing against this, there is little evidence of autoantibody deposition in and around infiltrated islets in type 1 diabetes. Moreover, in the majority of cases, the influent B-lymphocytes do not stain positively for the marker CD138 (21) which is recognized as being upregulated on antibody-producing plasma cells. Furthermore, the process of differentiation by which B-lymphocytes mature into plasma cells is usually associated with a loss of the surface marker CD20, but infiltrating islet-associated B-lymphocytes can be immunostained using an exogenously applied anti-CD20 antibody. Such evidence suggests very strongly that those B-lymphocytes present in and around inflamed islets are not engaged in autoantibody production. What is clear, is that the greater the number of B-lymphocytes in the islet inflammatory infiltrate, the fewer the number of beta-cells that remain at diagnosis, and the more likely a person is to have been diagnosed in the earliest years of life.

### The Role of Islet Infiltrating B-Lymphocytes: A Proposal

In view of these findings, we have begun to consider possible alternative roles for islet infiltrating B-lymphocytes in type 1 diabetes and have focused on the notion that they might be involved as potential determinants of CD8+ T-cell activity. It is increasingly understood that B-cells can exert a regulatory influence on T-cell activity (24) and there is firm support for the concept that B- and T-cell interactions occur within pancreatic lymph nodes (25). However, there has been much less focus on the possibility that such interactions may also take place within the islet milieu. To begin to explore this concept, we have examined the localization and morphology of CD20+ cells within the pancreas. This has revealed that significant morphological variations occur as B-lymphocytes migrate through the pancreas and that these correlate with their localization in relation to both CD8+ T-cells and the target islets. We have studied the disposition of CD20 on these cells since on the B-lymphocyte membrane this antigen is present at all stages of B-lymphocyte differentiation in humans, except during the very earliest phase and following their ultimate transition to plasma cells.

**Figure 1:**
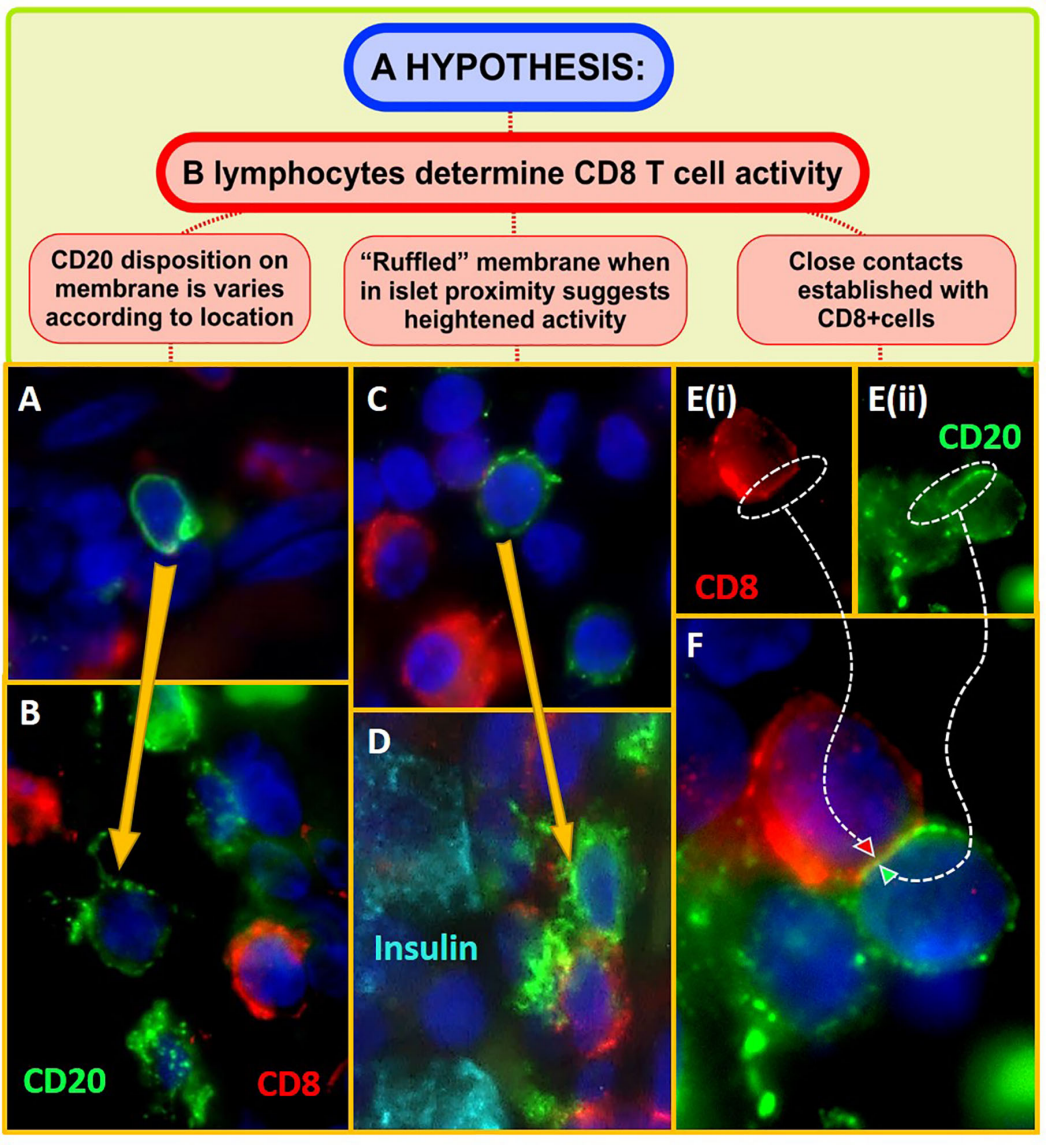
Micrographs showing CD20+ B-lymphocytes (green) and CD8+ T cells (red) within the pancreas of individuals with recent onset Type 1 diabetes. Sections of formalin fixed, paraffin embedded pancreas were dewaxed, rehydrated in an ethanol series, and subjected to heat-induced antigen retrieval (10mM citrate pH6) to unmask antigen binding sites. Highly specific and validated antibodies targeting CD20, CD8, and insulin (Dako, UK) were applied sequentially, in various combinations, for 1 h at room temperature, using standard immunofluorescence staining techniques. Positive signal was visualized with highly cross adsorbed Alexa Fluor-labeled secondary antibodies (Invitrogen, UK) along with the nuclear stain DAPI (dark blue; Invitrogen, UK). Micrographs were captured using either a Leica Dm4000 upright or a Leica DMi8 confocal microscope. **(A)** Depicts a CD20+cell in the pancreatic paranchyma, distant from an islet. The antigen is evenly distributed around the cell and the surface appears smooth in disposition. **(B)** When in proximity to an inflamed islet (i.e., when the CD20+cell was visible within the same region of interest as an islet), anti-CD20 molecules are seen to form aggregates on the cell surface. **(C, D)** Immunolabeling of CD20 becomes increasingly ruffled on the cell surface when the cells are located in closest proximity to insulin positive beta cells (cyan) and CD8+ T-cells. **(E, F)** shows a region of close apposition between a CD20+ B and CD8+ T lymphocyte. This is only seen when the cells are found in groups in close proximity to islets.

In the pancreas CD20+ B-lymphocytes can be found most often in close proximity to islets but some also reside at more distant locations within the parenchyma of the gland (**Figure 1**). Intriguingly, the arrangement of the surface CD20 antigen varies according to their localization within the gland and those B-lymphocytes found at greatest distances from islets (at least as evidenced by examination of 2D-sections) have a relatively uniform distribution of the CD20 antigen around their periphery (**Figure 1A**). This disposition changes, however, as the cells become more closely associated with islets, where they also begin to encounter CD8+ T-cells (**Figures 1B, C**). In these situations, the CD20 immunolabeling becomes focused in discrete regions of the plasma membrane, consistent with a possible change in the activation state of the cells. Moreover, in some cases, the membrane of the CD20+ cells shows intense ruffling and focusing of the antigen (**Figure 1D**). More strikingly still, as they arrive at the islet site and encounter increasing numbers of CD8+ T-cells, regions of close physical apposition can be discerned between neighboring CD8+ and CD20+ cells (**Figures 1E, F**). The formation of such sites of intimate contact may be important for sustaining and enhancing the state of CD8+ T-cell activation and could offer an explanation for the aggressive autoimmune attack and rapid loss of beta-cells, seen in certain individuals.

Evidence obtained in the NOD mouse has implied that B-cell depletion leads to reduced effector T-cell activation within inflamed islets (26) consistent with an inter-dependence in their actions and the present observations suggest that B- and CD8+ T-lymphocytes may also collaborate actively to promote beta-cell death in human type 1 diabetes.

**Figure 2:**
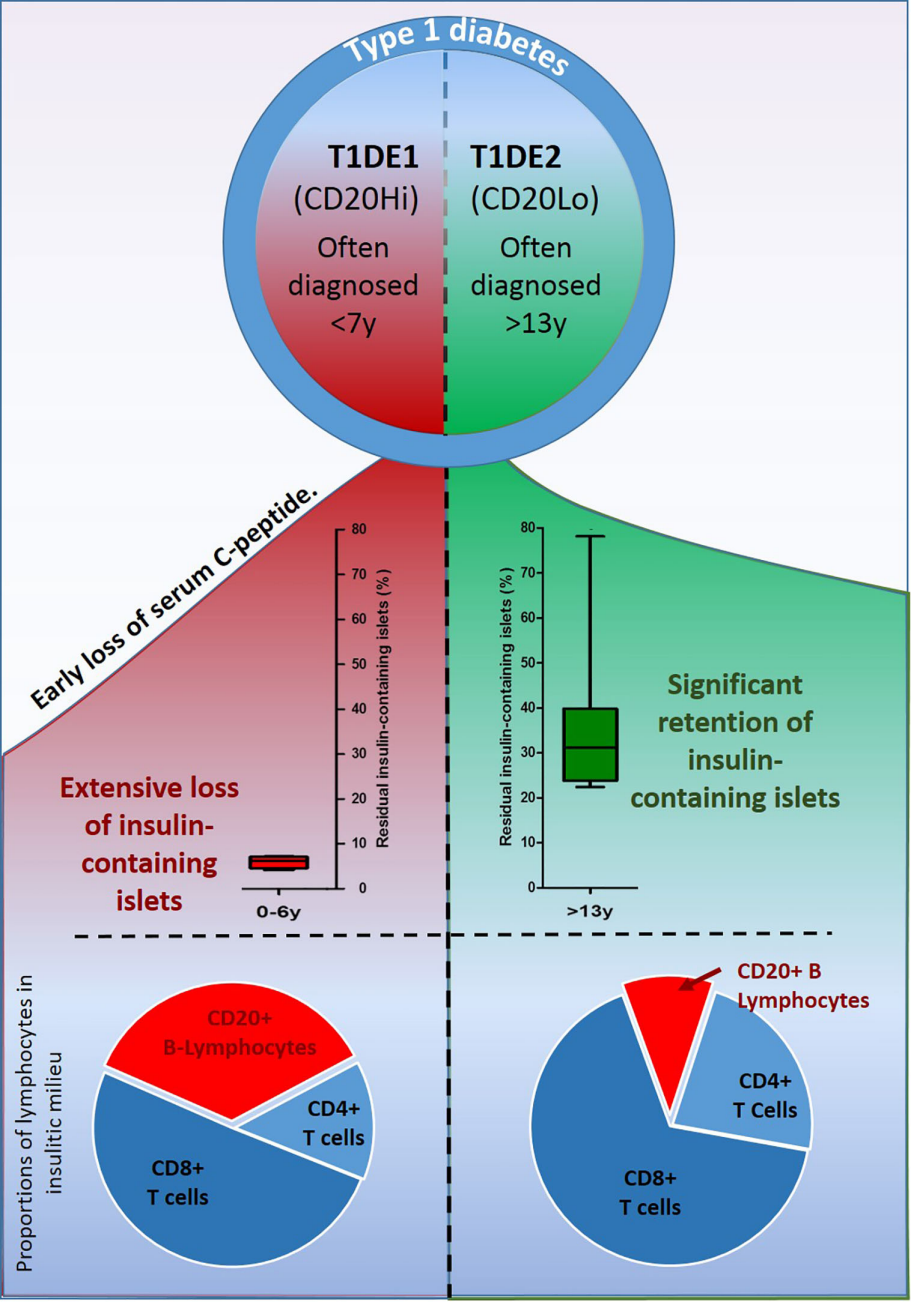
Schematic illustration depicting the main features differentiating the two recently described endotypes of type 1 diabetes. In T1DE1 (left panel) endogenous insulin production falls rapidly, few beta cells are found at diagnosis and symptoms of disease develop in the earliest years of life. B-lymphocytes comprise more than 35% of the immune cell population infiltrating inflamed islets. In T1DE2, subjects are more likely to retain detectable C-peptide well beyond diagnosis; a greater proportion of beta cells survive and clinical symptoms often develop during the teenage years. Individuals defined as T1DE2 have relatively few B-lymphocytes in inflamed islets at the time of diagnosis.

If this proposal has validity then it might also offer predictive capability; including, for example, the suggestion that, when the number of islet infiltrating B-lymphocytes are in the minority, the rate, and extent of beta cell loss should be correspondingly reduced. Precisely this situation is seen frequently in the inflamed islets of subjects diagnosed with type 1 diabetes during their teenage years (by contrast with those who are younger at onset and often display an inflammatory profile with an elevated proportion of B-lymphocytes) consistent with the existence of two disease endotypes. These have been recently termed “type 1 diabetes endotype 1” and “2” (abbreviated to T1DE1 and T1DE2) respectively (27). In both endotypes, the predominant immune cells are CD8+ T-cells, consistent with the view that these are likely to represent the principal effector population driving beta cell loss (**Figure 2**).

### How Might B-Lymphocytes Interact With CD8+ T-Cells in Islets?

An important question arising from these observations is the nature of the interaction between B- and CD8+ T-cells (as depicted in **Figures 1E, F**) and how this then leads to altered T-cell activation. One tempting possibility is to equate the establishment of the sites of most intimate contact as possible locations where “immune synapses” might form (28, 29). Immune synapses are short lived, planar structures which are extremely difficult to visualize within the confines of two dimensional fixed sections of pancreas. Nevertheless, the close apposition of the membranes of adjacent cells is consistent with this possibility and, as imaging modalities continue to evolve, this may prove a fertile area of exploration.

### What Are the Outcomes of Differential B-Lymphocyte Recruitment During Islet Inflammation?

Based on the proposition that CD20+ B-lymphocytes might play a role in promoting the activation state of CD8+ T-cells in and around inflamed islets, it should then be anticipated that beta cell loss would proceed least efficiently in situations where the proportion of B-lymphocytes is lowest (i.e. in subjects with T1DE2). While it cannot yet be deduced with certainty that this prediction is fulfilled (not least because the absolute number of CD8+ T-cells also varies substantially between individuals) the available evidence is consistent this possibility. This is most evident when considering two additional parameters which also differ between the two endotypes. Firstly, they segregate very strongly with age at diagnosis such that children with T1DE1 (where islet B-lymphocytes are present in greatest numbers) are diagnosed at the youngest ages and are often <7years at onset (27). By contrast, those with T1DE2 tend to be older when clinical symptoms arise and many are beyond 12 years of age. This difference does not, itself, imply that the rate of beta cell loss necessarily varies between children in each age group since the age at onset is likely to be influenced by many factors, including the specific age at which beta-cell destruction is initiated. Nevertheless, it is clear that the overall extent of beta-cell loss differs across age groups and that, at diagnosis, beta-cell loss is most profound in the youngest children (those under 7years). Not only so, but the number of residual beta cells present within the ICIs at onset is also reduced to a greater extent among the younger age group (**Figure 2**).

## Conclusion

In summary, we conclude by noting that, while autoantibody production by plasma cells may not be an absolute requirement in type 1 diabetes, this does not mean that B-lymphocytes play only a minimal role. Rather, islet-associated B-lymphocytes may play a profound role in influencing the outcome of autoimmunity in type 1 diabetes, perhaps by regulating the cytotoxic activity of their CD8+ T-cell counterparts. If true, then this has important implications therapeutically since it suggests that maneuvers designed to reduce the proportion of B-lymphocytes available within the islet milieu, may be effective in slowing the rate of beta-cell loss. It further suggests that such maneuvers are likely to be most effective at younger ages. Consistent with this, is the observation that administration of the anti-CD20 targeted monoclonal antibody, Rituximab, delayed disease onset most effectively in the younger sub-group among a cohort of subjects newly diagnosed with type 1 diabetes (30).

## Data Availability Statement

The original contributions presented in the study are included in the article/supplementary material. Further inquiries can be directed to the corresponding author.

## Author Contributions

PL conceived and performed experiments, developed experimental protocols, interpreted data, generated figures, and edited the manuscript. NM supervised the study, designed and interpreted experiments, and drafted the manuscript. All authors contributed to the article and approved the submitted version.

## Funding

We are grateful to Diabetes UK for financial support of this work *via* project grant 16/0005480 and to our many colleagues within the Exeter Centre for Excellence in Diabetes (ExCEED) for stimulating discussions.

## Conflict of Interest

The authors declare that the research was conducted in the absence of any commercial or financial relationships that could be construed as a potential conflict of interest.
